# Stroke Neurorehabilitation and the Role of Motor Imagery Training: Do ARAT and Barthel Index Improvements Support Its Clinical Use? A Systematic Review and Meta-Analysis

**DOI:** 10.3390/medicina62010174

**Published:** 2026-01-15

**Authors:** Luis Polo-Ferrero, Javier Torres-Alonso, Juan Luis Sánchez-González, Sara Hernández-Rubia, María Agudo Juan, Rubén Pérez-Elvira, Javier Oltra-Cucarella

**Affiliations:** 1 Department of Nursing and Physiotherapy, Universidad de Salamanca, 37007 Salamanca, Spain; javiertorres@usal.es (J.T.-A.); sarahernandezrubia@usal.es (S.H.-R.); 2 Instituto de Investigación Biomédica de Salamanca (IBSAL), 37007 Salamanca, Spain; juanluissanchez@usal.es; 3 Department of Medicine, Universidad de Salamanca, 37007 Salamanca, Spain; 4 Department of Psychobiology, Pontifical University of Salamanca, 37002 Salamanca, Spain; mjagudojuan@gmail.com (M.A.J.); rperezel@upsa.es (R.P.-E.); 5 Laboratory of Neuripsychophysiology, NEPSA Rehabilitación Neurológica, 37003 Salamanca, Spain; 6 Department of Health Psychology, Universidad Miguel Hernández de Elche, Avda. de la Universidad s/n, 03202 Elche, Spain

**Keywords:** stroke, Action Research Arm Test, Barthel Index, motor imagery, neurorehabilitation, meta-analysis, upper limb recovery

## Abstract

*Background and Objectives*: Although several meta-analyses have evaluated the effects of motor imagery (MI) on upper-limb recovery using the Fugl-Meyer Assessment for the Upper Extremity (FM-UE), evidence based on more specific (Action Research Arm Test, ARAT) and functional (Barthel Index, BI) outcomes remains scarce. This study examined the effect of MI combined with conventional rehabilitation therapy (CRT), which translates into meaningful improvements in upper-limb performance and functional independence after stroke, accounting for methodological quality and publication bias. *Materials and Methods:* A systematic review and meta-analysis were carried out in accordance with PRISMA recommendations, with prior registration in PROSPERO (CRD420251120044). Comprehensive searches were conducted across six electronic databases up to July 2025. The methodological rigor of the included studies was evaluated using the PEDro scale, and risk of bias was appraised with the Cochrane RoB 2 instrument. Random-effects models estimated pooled effect sizes (ESs) for the ARAT and BI, alongside analyses of heterogeneity, publication bias, and moderators. *Results*: Eleven RCTs (*n* = 425) were included. A small pooled improvement in ARAT was observed (ES = 0.25; 95% CI: 0.13–0.37; *p* < 0.001); however, this effect was rendered non-significant after correction for publication bias (ES = 0.08; 95% CI: −0.14–0.31). No significant differences were found for the BI (ES = 0.41; 95% CI: −0.35–1.18; *p* = 0.268), with substantial heterogeneity (I^2^ = 96.6%). The mean PEDro score was 6.6, indicating moderate methodological quality. *Conclusions*: MI combined with CRT yields small and inconsistent effects on upper-limb recovery and no improvement in functional independence. Current evidence does not support its routine use in stroke rehabilitation. Well-designed, adequately powered randomized controlled trials employing standardized MI protocols are required to determine its true clinical relevance.

## 1. Introduction

Stroke is one of the leading causes of long-term disability and mortality worldwide. Approximately 60% of stroke survivors continue to present upper-limb motor deficits six months after onset, including spasticity, abnormal movement synergies, weakness, and impaired dexterity and coordination, all of which substantially limit independence and quality of life [1,2,3]. Despite advances in neurorehabilitation, conventional physical and occupational therapies often fail to achieve full recovery, and only about 20% of stroke survivors regain complete motor function [4,5,6].

Upper-limb recovery after stroke has traditionally been quantified using impairment-based measures, most notably the Fugl-Meyer Assessment for the Upper Extremity (FM-UE), which provides a detailed evaluation of sensorimotor deficits and underlying neurophysiological recovery [7,8]. Although the FM-UE demonstrates excellent reliability and sensitivity to change, improvements at the impairment level do not necessarily translate into meaningful gains in functional hand use or independence in daily life. In this context, the Action Research Arm Test (ARAT) and the Barthel Index (BI) represent higher-level, clinically oriented outcomes. The ARAT assesses fine motor control, grasp, manipulation, and task-oriented upper-limb performance, whereas the BI reflects an individual’s ability to perform basic activities of daily living independently [9,10]. Consequently, demonstrating improvements in the ARAT and BI constitutes a more stringent test of clinical utility, as these measures reflect the real-world transfer of motor recovery into functional performance and autonomy—outcomes that ultimately determine rehabilitation success from the perspectives of patients, clinicians, and health-care systems [11].

Motor imagery (MI)—the cognitive rehearsal of movement without physical execution—has emerged as a promising adjunct to post-stroke rehabilitation [2,12]. MI activates neural circuits that overlap substantially with those engaged during actual movement execution, including the primary motor cortex, supplementary motor area, and parietal regions [13], supporting its theoretical potential to promote cortical reorganization and motor relearning. Given that repetitive and intensive task practice is a key driver of neuroplasticity and recovery [14], MI has been proposed as a low-cost and accessible strategy to complement conventional rehabilitation therapy (CRT). However, whether these neurophysiological effects translate into measurable improvements in dexterity, coordination, and functional independence remains uncertain.

Multiple randomized controlled trials (RCTs) have examined the effects of MI in post-stroke rehabilitation, reporting promising yet inconsistent findings. Although previous systematic reviews and meta-analyses have contributed useful evidence, their conclusions were limited by substantial methodological variability, small sample sizes, the inclusion of non-randomized designs, and the wide range of comparators employed, including conventional therapy, placebo conditions, and multimodal interventions. Comprehensive syntheses analyzing the ARAT and the BI were published up to late 2019 [15,16,17,18]. More recently, a rigorously conducted meta-analysis with advanced risk-of-bias assessment focused exclusively on impairment-level outcomes measured by the FM-UE and concluded that MI combined with CRT should not be routinely recommended to enhance motor recovery after stroke [19]. Thus, while prior work has clarified the effects of MI on motor impairment, whether MI-induced changes translate into meaningful improvements in upper-limb function and independence in daily life remains an unresolved research gap.

This gap is particularly relevant because only two prior meta-analyses evaluated the BI, both including the same three trials [17,18], highlighting the scarcity of quantitative evidence regarding functional independence and real-world outcomes. In addition, uncertainty persists regarding optimal MI training parameters, including frequency, duration, total dose, baseline motor severity, and recovery phase, due to the lack of standardized guidelines [20]. To date, only one randomized trial has systematically examined MI dosage, reporting a dose–response relationship for FM-UE improvements [21]. However, similar dose-dependent effects have not been consistently demonstrated for ARAT or BI outcomes, leaving open the question of whether impairment-level gains transfer to functional performance and autonomy.

Therefore, this meta-analysis aims to update and extend the existing evidence by systematically evaluating whether MI combined with CRT produces clinically meaningful improvements in upper-limb performance and functional independence after stroke, as assessed by the ARAT and the BI. We hypothesized that, despite reported effects on impairment-based outcomes, MI would not yield consistent or clinically relevant improvements in functional performance or independence when compared with CRT alone. To test this hypothesis, we applied stringent eligibility criteria and advanced analytical procedures, including sensitivity analyses, assessment of publication bias, and moderator analyses examining the influence of methodological quality, baseline motor severity, recovery phase, and intervention parameters.

## 2. Materials and Methods

### 2.1. Data Sources and Search Strategy

This systematic review was conducted in strict accordance with the Preferred Reporting Items for Systematic Reviews and Meta-Analyses (PRISMA) guidelines [22] and was prospectively registered in the International Prospective Register of Systematic Reviews (PROSPERO; registration ID: CRD420251120044, registration date: 5 August 2025).

A comprehensive and methodically structured search was undertaken to identify relevant studies. The electronic databases PubMed, Cochrane Library, CINAHL, Scopus, Web of Science, and ScienceDirect were systematically screened from their inception to July 2025. Search strategies were carefully tailored to the syntax and indexing systems of each database, employing controlled vocabulary terms (MeSH) and free-text keywords combined through Boolean operators to maximize sensitivity and specificity. The search was restricted to studies published in English. No restrictions were applied regarding publication date. Gray literature (e.g., theses, dissertations, and non-peer-reviewed reports) was not systematically searched. However, reference lists of all included studies were manually screened, and corresponding authors were contacted when clarification or missing data were required. The complete search algorithms and database-specific strategies are provided in Supplementary Material S1 to ensure transparency and reproducibility of the search process.

### 2.2. Eligibility Criteria

The inclusion criteria were established following the PICOS framework (Population, Intervention, Comparison, Outcomes, and Study Design) [23].

#### 2.2.1. Population

Studies were eligible if they included adult individuals with a confirmed stroke diagnosis who exhibited upper-limb functional impairment.

#### 2.2.2. Intervention

Eligible interventions consisted of MI combined with CRT.

#### 2.2.3. Comparison

Control groups were required to receive the same CRT administered to the intervention group, excluding the MI component.

#### 2.2.4. Outcomes

The Action Research Arm Test (ARAT) is a standardized assessment of upper-limb function and dexterity consisting of 19 items grouped into four subscales: gross grasp, grip, pinch, and gross arm movement, with a maximum score of 57 points [10]. Each item is scored from 0 to 3, with higher scores indicating better motor function. Total scores classify impairment severity as severe (≤24), moderate (25–44), mild (45–56), or normal [10]. The ARAT demonstrates excellent reliability (ICC = 0.96–0.99) [24], strong criterion validity through high correlation with the FM-UE (r = 0.93), and high sensitivity to detect clinical change [8].

The Barthel Index (BI) is a widely used scale for assessing functional ability in basic activities of daily living. It scores 10 activities, such as feeding, bathing, and mobility, on a scale up to 100 points, with higher scores indicating greater independence [9]. Scores are interpreted as: 100 (independent), 90–99 (minimal dependence), 60–89 (mild to moderate dependence), 40–59 (severe dependence), 20–39 (very severe dependence), and <20 (total dependence). The BI demonstrates excellent reliability and validity, with high sensitivity to change. In stroke patients, a modified version (MBI) showed a 0.95 correlation with the original BI, confirming its robustness for clinical practice and rehabilitation research [25]. The MBI maintains the same quantitative scoring system and interpretation thresholds as the original BI.

#### 2.2.5. Study Design

RCTs and pilot RCTs were included.

### 2.3. Study Selection

All records retrieved from the databases were imported into Rayyan software, where duplicate entries were automatically identified and removed prior to title and abstract screening. Two independent reviewers (LPF and JTA) screened all records using Rayyan software (https://www.rayyan.ai/, accessed on 15 July 2025)), adhering to a predefined selection protocol. Titles and abstracts identified through the database search were initially examined to exclude non-relevant publications. Subsequently, the full texts of potentially eligible studies were reviewed in detail to confirm their inclusion. Disagreements between reviewers were resolved through discussion and, when necessary, by consulting a third reviewer (JLSG) to reach consensus. Additionally, the reference lists of all included articles were manually searched to identify supplementary studies, and corresponding authors were contacted when clarification or missing data were required.

### 2.4. Data Extraction

Data extraction was performed independently by two reviewers using a standardized and pilot-tested data extraction form. Data extraction was conducted independently following a predefined protocol, and all collected information was cross-checked to guarantee completeness and accuracy.

For each eligible study, data were obtained regarding study design, sample size, and participant characteristics (age, sex, stroke phase, stroke type—ischemic or hemorrhagic—lesion laterality, and baseline severity based on ARAT and BI). Additional information included group allocation and detailed descriptions of the interventions, such as the type of MI, components of the conventional therapy, training frequency (sessions per week), session duration (minutes), and total intervention period (weeks). Reported outcomes and main study results were also documented.

When pre–post correlation coefficients were not reported, conservative imputed correlations were applied to calculate standardized mean changes. For ARAT outcomes, a correlation coefficient of 0.75 was assumed based on previously reported pre–post associations in stroke populations [26]. whereas a value of 0.80 was applied for BI and modified BI outcomes. The use of imputed pre–post correlations in this context is consistent with established methodological recommendations for pre–post controlled designs when within-group correlations are unavailable [27,28]. These imputed correlation coefficients were selected conservatively and are consistent with methodological guidance commonly applied in previous meta-analyses.

### 2.5. Risk of Bias and the Assessment of Methodological Quality of the Studies

The internal validity of the included trials was assessed using the revised Cochrane Risk of Bias tool for randomized studies (RoB 2), applying the specific version for crossover designs when required [29]. For parallel-group RCTs, this instrument evaluates five domains of potential bias: the randomization process, deviations from intended interventions, incomplete outcome data, measurement of outcomes, and selective reporting. Each study was categorized as having low risk of bias, some concerns, or high risk of bias. Two reviewers conducted the assessments independently, and inter-rater agreement was quantified using Cohen’s kappa for each domain as well as for the overall judgment [30]. Agreement strength was interpreted as follows: ≤0 no agreement, 0.01–0.20 slight, 0.21–0.40 fair, 0.41–0.60 moderate, 0.61–0.80 substantial, and 0.81–1.00 almost perfect agreement [31]. Any discrepancies were resolved through discussion with a third reviewer until full consensus was reached.

Methodological quality was additionally evaluated using the Physiotherapy Evidence Database (PEDro) scale [32], which assesses both internal and external validity across 11 items: (1) clearly stated eligibility criteria; (2) random allocation; (3) concealed allocation; (4) baseline comparability; (5) blinding of participants; (6) blinding of therapists; (7) blinding of assessors; (8) attrition < 15%; (9) intention-to-treat analysis; (10) between-group statistical comparisons; and (11) reporting of point estimates with variability measures. Although item (1) is required for completeness, it is not included in the overall PEDro score. Each criterion was rated as “yes”, “no”, or “unclear”.

The PEDro ratings served as a complement to the RoB 2 evaluations, offering an additional indication of methodological soundness. Based on total PEDro scores, studies were classified as excellent (9–10), good (6–8), fair (4–5), or poor (0–3) [33].

### 2.6. Studies Data Synthesis and Analysis

The quantitative synthesis was conducted using R statistical software (R Foundation for Statistical Computing, Vienna, Austria; version 2024.12.0.467) [34]. The primary effect size (ES) of interest represented the pre–post change in each study’s main outcome when comparing the intervention and control groups. All analyses were carried out with the metafor package [35], applying a multivariate random-effects model with a random intercept for each study via the meta() function. Sensitivity analyses were conducted to assess the robustness of the pooled estimates to the imputed pre–post correlation assumptions.

Standardized Mean Change (SMC) values based on raw scores were computed using the escalc() function, following the formula $(M_{post}-M_{pre})/SD_{pre}$, where $M_{pre}$ and $M_{post}$ denote baseline and post-intervention means, respectively, and $SD_{pre}$ is the standard deviation at baseline. Because the variance of the SMC depends on the correlation between pre- and post-test scores, correlations were directly calculated from available primary data; when not reported, conservative imputed values were applied. Specifically, a correlation coefficient of r = 0.75 was used for the ARAT [26], whereas a coefficient of r = 0.80 was applied for the BI and the MBI, given that no pre–post correlations were identified for these outcomes and this value has been recommended in previous methodological works [27].

To maintain a uniform interpretation of effects, ESs were reversed when needed so that positive values consistently indicated improvement due to the intervention. A multilevel model was then fitted using the rma.mv() function, incorporating a random intercept for each study and estimating heterogeneity through restricted maximum likelihood (REML). After deriving ESs for all groups, a single pooled ES per study was obtained using the aggregate() function, applying the variance–covariance structure generated in the earlier step.

Effect sizes were weighted by the inverse of their total variance, accounting for sampling variance, between-study variance (τ^2^), and within-study covariance among effects [36]. Consistent with conventional thresholds, overall ES magnitudes were classified as small (0.20–0.49), moderate (0.50–0.79), or large (≥0.80) [37].

#### 2.6.1. Heterogeneity in ES Estimates

Between-study variability was quantified using the orchaRd package [38]. The I^2^ statistic quantified the proportion of total variability attributable to true heterogeneity rather than sampling error [39], with thresholds of 25%, 50%, and 75% interpreted as low, moderate, and high heterogeneity, respectively [40].

#### 2.6.2. Sensitivity Analysis

A leave-one-out sensitivity procedure was conducted using the leave_one_out() function from the orchaRd package to evaluate the stability of the pooled effect size [38]. In this approach, each study was omitted individually, and the resulting changes in the overall effect estimate were assessed. The corresponding outputs were displayed using the orchard_leave1out() visualization function.

#### 2.6.3. Publication Bias Analysis

Potential publication bias was evaluated using the pub_bias_plot() function from the orchaRd package [38], which estimates changes in the overall effect size attributable to selective reporting. The Publication Bias analysis was performed in three steps: (1) first, we fitted a fixed-effects model with the data obtained with the escalc() function; (2) second, a robust variance estimation was calculated with the robust() function to account for within-study dependence of multiple effect sizes; and (3) third, we conducting a multilevel Egger’s regression test in which the sampling variance of each effect size was modeled as a predictor [41]. The resulting changes in the overall effect size across models were graphically represented with the pub_bias_plot() function.

#### 2.6.4. Moderator Analyses

In order to examine the influence of potential moderators on the pooled effect size, a series of univariate meta-regressions was conducted including mean participant age, sex distribution, PEDro score, risk of bias classification, stroke phase (using the subacute phase as the reference), intervention frequency, total number of sessions, and session duration. For analyses involving the risk of bias scale, studies with low risk served as the reference category, based on prior evidence indicating that trials with higher bias levels may overestimate effect sizes [42,43]. All effect size estimations were computed using *t*-distributions for coefficients and confidence intervals. The complete analysis code can be accessed from the authors’ website [44].

## 3. Results

### 3.1. Search Outcome and Study Inclusion Process

The literature search yielded a total of 4074 records across the selected databases (PubMed, *n* = 581; Cochrane Library, *n* = 507; ScienceDirect, *n* = 756; Scopus, *n* = 846; Web of Science, *n* = 1113; and CINAHL, *n* = 271). After removing 1684 duplicate records, 2390 titles and abstracts were screened for eligibility. Of these, 2187 records were excluded for not meeting the predefined inclusion criteria.

**Figure 1:**
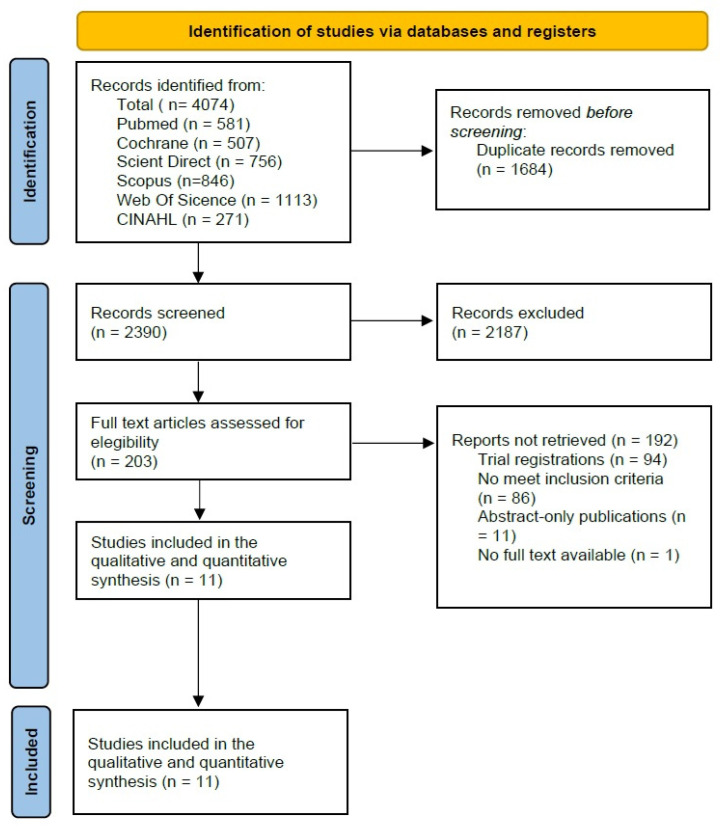
Prisma 2020 Flow Diagram [22].

Subsequently, 203 full-text articles were assessed in detail, leading to the exclusion of 193 studies: 94 corresponded to study registry entries, 86 did not meet the inclusion criteria, 11 were conference abstracts, and one study lacked accessible full text. Ultimately, 11 RCTs were included in the systematic review and meta-analysis. A detailed overview of the study selection process is presented in the PRISMA flow diagram (Figure 1).

### 3.2. Study Characteristics

A total of 425 participants were included across the 11 studies, comprising 204 individuals in the IG, 182 in the CG, and 39 receiving placebo. One study did not report participants’ age or sex distribution [45]. The mean age in the IG was 58.6 ± 5.5 years (32.0 ± 18.0% women), while the CG had a mean age of 60.1 ± 5.8 years (34.0 ± 21.0% women). All studies were randomized controlled trials, with one identified by its authors as a pilot RCT [45]. The mean sample size per group was 17.6 ± 9.8 participants in the IG and 16.3 ± 8.7 in the CG.

Most intervention groups were classified as having severe baseline upper-limb impairment according to the ARAT (*n* = 4 studies) [45,46,47,48], followed by moderate impairment (*n* = 2 studies) [49,50], and mild deficits (*n* = 1 study) [51]. Baseline functional dependence, assessed using the BI or MBI, showed a heterogeneous distribution across trials. Two studies reported total dependence in both groups [49,52], while two others included participants with mild to moderate dependence [45,51]. In the remaining four studies, the IG presented greater functional dependence at baseline (severe dependence) compared with the CG, which exhibited mild to moderate dependence. This pattern was observed in two groups from Fuzail [45], as well as in three other studies [53,54,55].

In terms of stroke etiology, one study included only ischemic stroke patients [46], four studies involved both ischemic and hemorrhagic strokes [47,53,54,55], and three provided detailed breakdowns by type, reporting 8.0 ± 1.0 ischemic cases and 6.7 ± 1.5 hemorrhagic strokes in CG and 6.7 ± 2.1 ischemic cases and 8.7 ± 5.1 hemorrhagic cases in the IG [53,54,55]. Lesion laterality was reported in all studies except two [45,50]. When provided, both hemispheres were affected across samples, showing a balanced distribution (left: 7.9 ± 5.2; right: 7.9 ± 3.9 participants). Concerning stroke chronicity, five studies were conducted in the subacute phase [46,48,49,52,55], five in the chronic phase [45,47,51,53,54], and one included both subacute and chronic patients [50].

Intervention parameters varied across studies. The mean intervention duration was 4.0 ± 1.4 weeks for both groups, except for one study [52], which prescribed a minimum of 10 sessions rather than a defined time frame. The total study duration ranged from a single session [45] to seven weeks [48]. The mean training frequency was 4.4 ± 1.9 sessions per week, yielding an average of 14.9 ± 6.6 sessions per participant. CRT lasted 68.0 ± 62.9 min per session and was comparable between groups. The MI was not specified in one study [52]; however, the remaining trials reported an average MI time of 39.0 ± 13.4 min per session, corresponding to a cumulative exposure of 503.5 ± 375.4 min per participant. Further details on study characteristics and intervention parameters are summarized in Table 1.

### 3.3. Methodological Quality

The methodological quality of the eleven included trials was moderate, with a mean PEDro score = 6.6 points. Specifically, four studies obtained 5 points [45,51,53,55], another one scored 6 points [46], three reached 7 points [47,48,50], two scored 8 points [49,52] and one scored 9 points [54]. The main methodological limitations were the absence of concealed allocation and participant or therapist blinding, which was not achieved in any trial. Although assessor blinding was common, adherence to intention-to-treat analysis and retention above 85% was inconsistent. Overall, while the RCTs met basic internal validity standards, weaknesses in allocation concealment and blinding remain the most evident sources of potential bias. Detailed PEDro scores are shown in Table 2.

### 3.4. Risk of Bias

**Figure 2:**
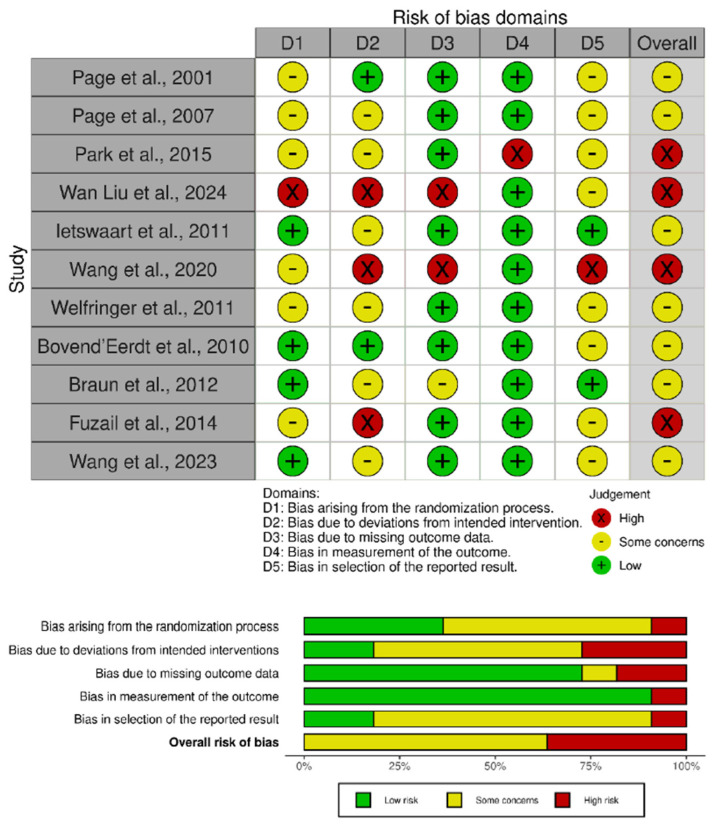
Risk of bias assessment for the included studies [45,46,47,48,49,50,51,52,53,54,55].

The methodological quality of the included trials was evaluated using the Cochrane RoB 2 tool for randomized parallel designs. A summary of the overall judgments is presented in Figure 2. The agreement between reviewers was high prior to consensus (Cohen’s κ = 0.82), indicating strong consistency in the assessments. Seven studies were classified as having “Some Concerns” of bias [46,47,48,49,50,52,54]. These concerns were mainly associated with insufficient reporting of the randomization and allocation concealment processes, as well as incomplete information in the domain of selective reporting. Four trials were considered at “High Risk” of bias [45,51,53,55], primarily due to deviations from intended interventions and incomplete outcome data. In several of these studies, the lack of participant or assessor blinding and the absence of intention-to-treat analyses likely contributed to performance and attrition bias.

### 3.5. Evaluation of MI Effects on ARAT Performance

#### 3.5.1. Meta-Analytic Findings for MI Effects on ARAT

**Figure 3:**
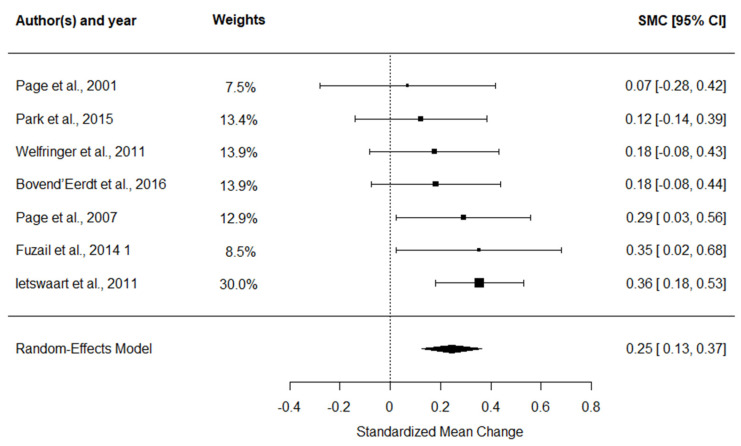
Forest plot of SMC with 95% confidence intervals for the effect of MI combined with CRT on ARAT performance [45,46,47,48,49,50,51].

The ARAT meta-analysis (Figure 3) indicated a small overall ES of 0.25 (95% CI: 0.13 to 0.37, *p* < 0.001), with no observed heterogeneity (I^2^ = 0%).

#### 3.5.2. Sensitivity Analyses on ARAT

**Figure 4:**
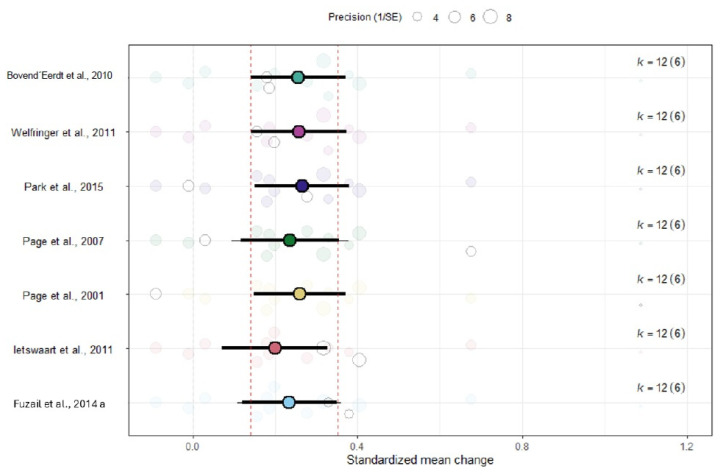
Leave-one-out sensitivity analysis assessing the robustness of the pooled ARAT effect size [45,46,47,48,49,50,51].

The sensitivity analysis showed that the ES remained stable after removing one study at a time (Figure 4), which is in line with the absence of heterogeneity between ESs among studies.

#### 3.5.3. Publication Bias Assessment on ARAT

**Figure 5:**
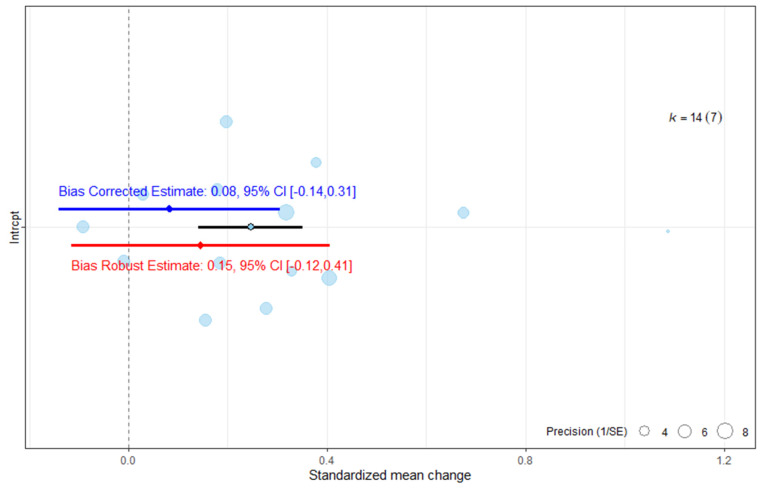
Publication bias analysis of overall effect size for the ARAT. The blue line represents the bias-corrected estimate, while the red line represents the bias-robust estimate; horizontal lines indicate the corresponding 95% CI.

The publication bias assessment indicated that the ES would no longer reach statistical significance once publication bias was accounted for (Figure 5). The robust estimate likewise lost statistical significance.

### 3.6. Analysis of the Effects of MI on Functional Independence

#### 3.6.1. Meta-Analytic Findings for MI Effects on BI

**Figure 6:**
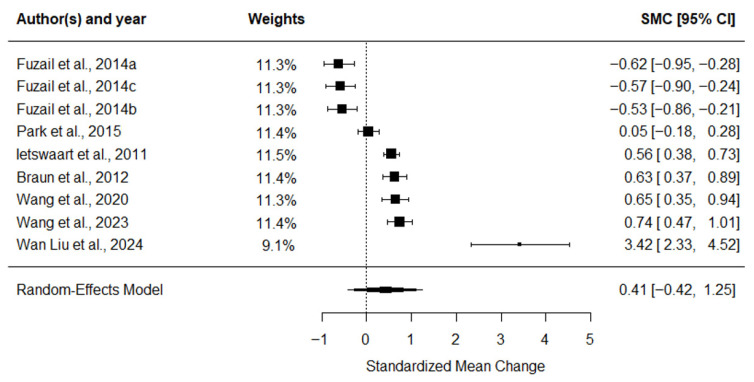
Forest plot of SMC with 95% confidence intervals for the effect of MI combined with CRT on functional independence assessed by the BI [45,49,51,52,53,54,55].

The BI meta-analysis (Figure 6) yielded a small-to-moderate ES of 0.41 (95% CI: −0.35 to 1.18, *p* = 0.268), which was not statistically significant and showed substantial heterogeneity (I^2^ = 96.6%).

#### 3.6.2. Sensitivity Analyses on Functional Independence

**Figure 7:**
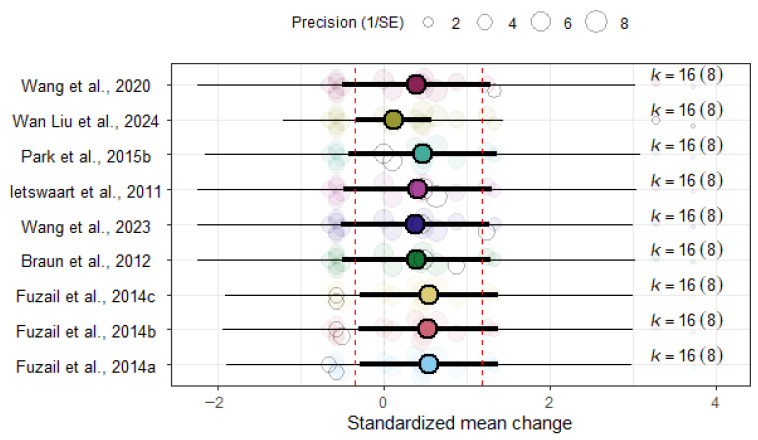
Leave-one-out sensitivity analysis for the overall BI effect size [45,49,51,52,53,54,55].

The sensitivity analysis showed that the ES remained stable after removing one study at a time (Figure 7). These results must be interpreted in line with the high heterogeneity between ESs among studies and the results in Figure 6, which shows ESs ranging from −0.62 to 3.42, and suggests that moderator analysis is necessary in this context.

#### 3.6.3. Publication Bias Assessment on Functional Independence

**Figure 8:**
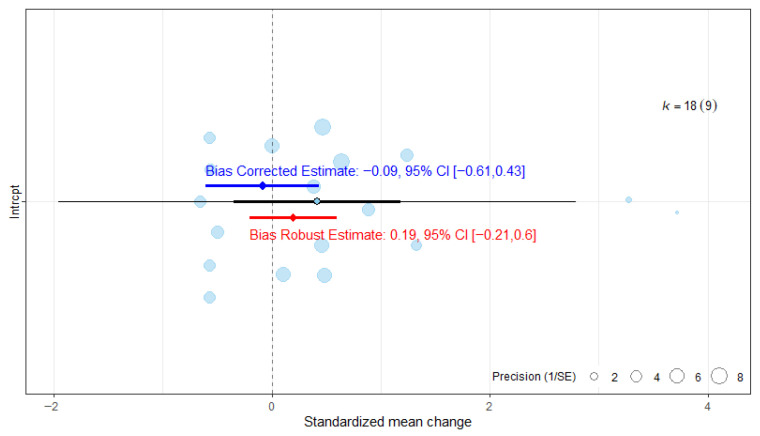
Publication bias analysis of overall effect size for the Barthel Index. The blue line represents the bias-corrected estimate, while the red line represents the bias-robust estimate; horizontal lines indicate the corresponding 95% CI.

The assessment of publication bias indicated that the ES would continue to be statistically non-significant once publication bias was accounted for (Figure 8). The robust estimate likewise remained non-significant.

### 3.7. Moderator Analyses

The overall ES for the ARAT was not associated with sample size (b = 0.01, *p* = 0.175), age (b = 0.01, *p* = 0.383), sex (b = 0.482, *p* = 0.396), the RoB score (b = −0.04, *p* = 0.728), the PEDro score (b = 0.05, *p* = 0.280), the phase of the disease (b = −0.07, *p* = 0.255), the total intervention time (b = 0.00, *p* = 0.548), the total number of sessions (b = 0.00, *p* = 0.785) or the time of MI sessions (*p*’s > 0.089).

For the BI no significant associations were observed between the overall effect size and sample size (b = 0.04, *p* = 0.089), age (b = −0.05, *p* = 0.074), sex (b = −0.92, *p* = 0.388), the RoB score (b = −0.33, *p* = 0.693), the PEDro score (b = 0.10, *p* = 0.677), phase of the disease (b = −0.33, *p* = 0.687), the total intervention time (b = 0.00, = 0.051), total number of sessions (b = 0.02, *p* = 0.219) or the time of MI sessions (*p*’s > 0.230).

## 4. Discussion

This meta-analysis synthesized evidence from 11 RCTs evaluating the effects of MI combined with CRT on upper limb recovery and functional independence after stroke. The results revealed a small positive effect of MI on upper limb performance, as measured by the ARAT, but this effect became statistically non-significant after adjusting for publication bias and applying robust variance estimation. No significant improvements were observed in functional independence assessed by the BI, and heterogeneity among studies was high.

The divergent patterns observed for ARAT and BI outcomes warrant explicit consideration. The ARAT yielded a small but homogeneous effect, whereas BI results were highly heterogeneous and non-significant. This discrepancy likely reflects fundamental differences in outcome specificity. The ARAT is a task-oriented measure designed to capture upper-limb dexterity, grasp, and fine motor control—domains directly targeted by MI interventions [10]. In contrast, the BI assesses global functional independence across multiple domains, including mobility, transfers, continence, and self-care, many of which are only partially dependent on upper-limb function [9]. Consequently, improvements in hand performance may not translate into detectable changes in overall independence, given the multidimensional nature of global disability measures [7]. Moreover, substantial between-study variability in baseline BI scores, baseline imbalances between intervention and control groups, and differences in care settings and CRT content are likely to have amplified heterogeneity and obscured any small additive effect of MI on BI outcomes.

These findings contrast with previous quantitative syntheses that reported modest but significant improvements in ARAT scores [15,16,17,18]. The present results suggest that earlier conclusions may have been overestimated due to publication bias. When the selective reporting of positive outcomes was modeled, the adjusted overall effect not only decreased but reversed, indicating marked asymmetry in the literature—small studies with favorable results are more likely to be published, whereas null or negative trials remain underrepresented. Regarding functional independence (BI), despite including a greater number of trials in the present analysis, no significant effects emerged—neither before nor after correction for publication bias—consistent with prior evidence showing the absence of measurable improvements in activities of daily living [17,18]. Similarly, these findings align with meta-analyses based on the FM-UE, where apparent benefits of MI also disappeared after accounting for reporting bias [19].

Taken together, the available evidence suggests that combining MI with CRT does not result in consistent or clinically meaningful improvements in upper-limb function or functional independence after stroke. Although small benefits have been reported in some studies, these effects appear to be strongly influenced by selective reporting and methodological limitations, reinforcing that neural activation alone does not necessarily translate into improved dexterity or independence [56,57]. Consequently, caution is warranted when considering MI as a routine adjunct in clinical practice.

When interpreting the clinical significance of the observed effects, it is important to consider the Minimal Clinically Important Difference (MCID) values for the ARAT, which range between 12 and 17 points for subacute patients [58] and approximately 5.7–6 points for chronic stroke survivors [59]. According to these thresholds, only two studies in the present meta-analysis reported mean changes exceeding the MCID—one in participants with severe and another in those with moderate impairment [46,47]—suggesting that most interventions combining MI with CRT fail to achieve clinically meaningful improvement beyond measurement error. This finding may reflect a ceiling effect in patients with mild-to-moderate impairment, whose limited potential for improvement constrains measurable change, whereas individuals with greater baseline deficits possess a larger recovery margin, disproportionately influencing pooled estimates [60,61].

Despite exploring several potential moderators—including demographic factors, methodological quality, stroke chronicity, and intervention parameters—none showed a statistically significant association with overall effect sizes for either the ARAT or the BI. These results suggest that heterogeneity cannot be attributed to differences in participant characteristics (age, sex), study design (sample size, PEDro score, or risk of bias), or intervention dose (number, duration, or total session time). Similarly, previous analyses based on the FM-UE have also failed to identify significant moderating effects related to these variables, reinforcing that variability in MI outcomes is not systematically explained by demographic, methodological, or intervention-related factors [19].

An additional limitation relates to the clinical heterogeneity of the included populations. The ARAT is primarily designed to assess task-oriented upper-limb performance in individuals with mild-to-moderate motor impairment, and its sensitivity may be reduced in patients with more severe paresis [9,10]. Accordingly, variability in baseline motor severity across trials—often reflected in whether ARAT was used as a standalone outcome or alongside impairment-based measures such as the FM-UE—may have contributed to between-study heterogeneity [7,19]. Importantly, baseline motor severity was explored in sensitivity analyses and did not emerge as a significant moderator of effect size; however, this finding should be interpreted with caution given the limited number of available studies and the resulting low statistical power to detect subgroup effects. Although in ARAT and BI scores were observed in some trials, sensitivity analyses did not indicate a disproportionate influence of these studies on the pooled results; however, excluding them would have substantially reduced the number of available studies and statistical power. Taken together, these considerations should be accounted for when interpreting pooled estimates, while the overall conclusion remains that MI-related gains do not consistently translate into clinically meaningful functional or independence-related improvements.

Moreover, key parameters of MI—including session length, weekly dosage, total practice time, and the type of guidance provided—showed substantial variability across the included trials, as did stroke chronicity and baseline motor deficits. Important procedural aspects, such as whether imagined movements were transitive or intransitive, the use of rest intervals, or strategies to sustain attention and imagery vividness, were typically underreported. This methodological imprecision reduces interpretability and makes it difficult to determine the most effective MI parameters, optimal dosage, or the recovery stage in which MI may be most beneficial. Although one study reported a possible dose–response pattern suggesting that longer MI exposure might enhance ARAT outcomes [21], this effect was not statistically significant and aligns with our findings. The absence of differential effects across stroke phases may also relate to the persistence of neuroplastic potential well beyond the acute period [62]. Furthermore, limited reporting of stroke recurrence may have contributed to clinical variability, as recurrent strokes often entail more severe neurological involvement and slower recovery profiles.

A further limitation involves the heterogeneity in CRT implementation. The specific components of CRT varied notably across studies, and inconsistencies in what was considered “conventional rehabilitation” may have influenced outcomes. Future clinical trials should provide clearer descriptions of CRT content; however, the present analysis was designed to isolate the added value of MI irrespective of CRT variation. Overall, substantial variability in intervention protocols, small sample sizes, short treatment durations, and absence of follow-up data underscore the need for more rigorous and standardized research designs. Well-powered RCTs and consensus on MI dosing—frequency, intensity, duration, and total exposure—are essential to improve reproducibility and enhance clinical relevance. Another important limitation is the lack of formal assessment of MI ability, reported in only two trials [46,51]. Given that imagery capacity is frequently reduced after stroke and directly influences the effectiveness of MI interventions [63,64], failing to evaluate it may have attenuated true effects. Future studies should routinely screen imagery ability using validated instruments to ensure appropriate participant selection.

Advancing MI research will require stronger methodological consistency and standardization. Developing unified MI protocols with clearly defined frequency, dose, duration, and total training volume will support comparability across trials. Baseline evaluation of MI capacity should be incorporated to include only participants capable of generating accurate MI. Larger RCTs with extended follow-up are necessary to verify long-term benefits and identify subgroups that may respond preferentially. Additionally, future research should systematically incorporate the FM-UE for motor impairment, the ARAT for fine motor and grasp performance, and the BI for functional independence, allowing for a more integrated evaluation of MI’s impact. Transparent reporting of null or negative results remains crucial to mitigate publication bias and strengthen the evidence base.

Given the weak and inconsistent functional effects observed for MI when delivered as a standalone adjunct to CRT, emerging technologies may be required to augment MI to a level capable of producing clinically meaningful functional benefits. Combining MI with virtual or augmented reality may help intensify and contextualize motor network engagement and facilitate the transition from imagined to executed movement [65,66]. Likewise, integrating MI with closed-loop brain–computer interfaces, functional electrical stimulation (BCI–FES), or robotic systems could potentiate neuroplastic mechanisms beyond what is achievable with conventional MI delivery [67,68]. Similarly, pairing MI with non-invasive brain stimulation—such as transcranial direct current stimulation (tDCS) or repetitive transcranial magnetic stimulation (rTMS)—may further enhance cortical excitability and motor relearning [11].

Less than 40% of stroke survivors regain useful upper-limb function, underscoring the limitations of current rehabilitation [4,69]. Although MI combined with CRT remains theoretically appealing, its clinical efficacy remains uncertain. Therapist-guided, task-specific MI embedded within CRT—and applied to individuals with adequate cognitive and imagery ability—may offer the greatest potential. Overall, optimizing MI integration will require methodological standardization, careful participant selection, and innovation in intervention design. The incorporation of validated outcome metrics and emerging neurorehabilitation technologies may refine MI delivery, enhance neural activation, and ultimately support improved functional recovery.

## 5. Conclusions

MI combined with CRT was associated with a small improvement in upper-limb function as measured by the ARAT; however, this effect became non-significant after correction for publication bias, limiting the robustness of the evidence. Moreover, the magnitude of the observed effect is unlikely to reach clinically meaningful thresholds for upper-limb function as defined by established ARAT MCID values. No significant effects were observed for functional independence assessed by the BI, and the substantial heterogeneity across studies suggests inconsistent findings. Taken together, current evidence does not consistently support a clinically relevant benefit of MI for motor or functional recovery after stroke, highlighting the need for larger, methodologically rigorous trials to better define its potential role, target populations, and optimal implementation parameters.

## Figures and Tables

**Table 1:** Baseline Characteristics.

Study	Study Design	Phase Stroke	Etiology	Lesion Laterality	Groups	Age	Intervention	Intervention Volume			Outcomes	Results
								Weeks	Frequency	Session Duration (Minutes)		
Braun et al., 2012 [52]	RCT	Subacute	-	Both	IG (18)	77.5	Multi-professional rehabilitation + mental practice techniques	6	Min. 10	-	MI BI 9HPT BBS RMI 10MWT	Patients in both groups significantly improved on almost all outcome measures, but the differences in improvements between the 2 groups were small.
					CG (18)	77.9	Multi-professional rehabilitation	6	Min 10	-		
Fuzail et al., 2014 [45]	Pilot RCT	Chronic	-	-	IG (10)	-	Single-task training with auditory imagery techniques	1	1	45	ARAT MAL-AOU MAL-QOM BI	MI significantly improved upper limb function in chronic stroke patients compared to physiotherapy alone.
					IG (10)	-	Single-task training with visual imagery techniques	1	1	45		
					IG (10)	-	Single-task training with auditory and visual techniques	1	1	45		
					CG (10)	-	Single task training	1	1	45		
Wang et al., 2023 [54]	RCT	Chronic	Ischemic and hemorrhagic	Both	IG (17)	54.36	CRT + supervised MI (relaxation, basic movements, goal-directed daily tasks)	4	5	MI: 30 CRT: 180	FM-UE MBI fMRI	Participants receiving MI showed greater FM-UE improvements than CG, accompanied by changes in slow-5 fractional amplitude of low-frequency fluctuations and ipsilesional inferior parietal connectivity, neuroimaging markers previously associated with motor improvement.
					CG (17)	59.71	CRT	4	5	180		
Ietswaart et al., 2011 [49]	RCT	Subacute	-	Both	IG (41)	69.3	Structured MI training focused on the affected upper limb, including imagined elementary movements, goal-directed tasks, activities of daily living, and facilitated imagery and CRT	4	5	MI: 45 CRT: 40	ARAT HG Hand function BI	MP with MI showed no significant effects on motor recovery in early post-stroke patients.
					Placebo (39)	68.6	Visual and sensory IM not related to motor control	4	5	45		
					CG (41)	64.4	CRT	4	5	40		
Park et al., 2015 [51]	RCT	Chronic	ni	Both	IG (14)	60	MI centered on daily task practice (page turning, bean transfer, cup stacking) combined with CRT.	2	5	MI:10 CRT: 30	FM-UE ARAT MBI	MI enhanced upper-limb function and performance in daily activities.
					CG (15)	58	CRT	2	5	40		
Wan Liu et al., 2024 [55]	RCT	Subacute	Ischemic and hemorrhagic	Both	IG (13)	58.63	CRT (physiotherapy, occupational therapy, electrical stimulation, Chinese acupuncture) combined with targeted MI training.	4	5	IM: 30 CRT: 120	MBI FM-UE	MI combined with CRT led to greater improvements in upper-limb function and daily activities than CRT alone.
					CG (13)	60.17	CRT (physical therapy, occupational therapy, electrical stimulation, and Chinese acupuncture)	4	5	120		
Wang et al., 2020 [53]		Chronic	Ischemic and hemorrhagic	Both	IG (17)	53.38	CRT + supervised MI for the affected upper limb (relaxation, basic movements, goal-directed daily tasks)	4	5	IM: 30 CRT: 180	FM-UE MBI fMRI	MI improved FM-UE more than CG and produced increases in slow-5 fractional amplitude of low-frequency fluctuations and changes in ipsilesional inferior parietal connectivity, both linked to motor recovery.
					CG (17)	60.47	CRT	4	5	180		
Page et al., 2001 [46]	RCT	Subacute			IG (8)	64.4	CRT with upper/lower limb exercises, transfers, balance and gait training, and bimanual ADL practice, plus guided MI sessions after each therapy.	6	3	MI: 10 CRT: 60	FM-UE ARAT	MI was shown to be a feasible and low-resource adjunct to CRT, with modest improvements observed relative to therapy alone.
					CG (5)	65.0	CRT consisting of the same upper- and lower-limb exercises, transfer training, balance/walking activities, and ADL routines.	6	3	60		
Page et al., 2007 [47]	RCT	Chronic			IG (16)	58.7	CRT focused on activities of daily living, combined with daily MP sessions directly after therapy.	6	2	MI: 30 CRT: 30	FM-UE ARAT	MP enhanced arm motor function in individuals with chronic stroke.
					CG (16)	60.4	CRT with equal therapist interaction.	6	2	30		
Welfringer et al., 2011 [48]	RCT	Subacute			IG (15)	56.3	CRT supplemented with visuomotor MI therapy of the contralesional upper limb involving repetitive practice of positions and movement sequences plus CRT	3	MI: 7 CRT: 4	MI: 60 CRT: 45	BCT AFTS Representation tests Body touching ARAT	Kinesthetic visuomotor MI therapy was feasible and improved body and space perception in subacute neglect patients.
					CG (15)	57.1	CRT	3	4			
Bovend’Eerdt 2010 [50]	RCT	Subacute and Chronic			IG (15)	51.2	CRT (standard physiotherapy and occupational therapy) supplemented with MI practice	6	3	MI:10 CRT: 50	GAS RMI NEADL ARAT TUG	Both groups improved over time, with no significant differences between integrated MI and RCT.
					CG (15)	50.6	CRT (standard physiotherapy and occupational therapy)	6	3	50		

Abbreviations: 10MWT, Ten-Meter Walking Test; 9HPT, Nine-Hole Peg Test; AFTS, Arm Function Test Sensation; ARAT, Action Research Arm Test; BBS, Berg Balance Scale; BCT, Bells Cancelation Test; BI, Barthel Index; CG, Control Group; CRT, Conventional Rehabilitation Therapy; FM-UE, Fugl-Meyer Upper Extremity; fMRI, Functional Magnetic Resonance Imaging; GAS, Goal Attainment Scaling; HG, Hand Grip; IG, Intervention Group; MAL-AOU, Motor Activity Log-Amount of Use; MAL-QOM, Motor Activity Log-Quality of Movement; MBI, Modified Barthel Index;; MI, Motor Imagery; MP, Mental Practice; NEADL, Nottingham Extended Activities of Daily Living; ni, no information; RCT, Randomized Clinical Trial; RMI, Rivermead Mobility Index;; TUG, Timed Up and Go.

**Table 2:** Methodological quality of the RCTs assessed with the PEDro scale.

Study	1	2	3	4	5	6	7	8	9	10	11	Total
Fuzail et al., 2014 [45]	Y	Y	N	Y	N	N	Y	N	N	Y	Y	5
Ietswaart et al., 2011 [49]	Y	Y	Y	Y	N	N	Y	Y	Y	Y	Y	8
Page et al., 2001 [46]	Y	Y	N	N	N	N	Y	Y	Y	Y	Y	6
Page et al., 2007 [47]	Y	Y	N	Y	N	N	Y	Y	Y	Y	Y	7
Welfringer et al., 2011 [48]	Y	Y	N	Y	N	N	Y	Y	Y	Y	Y	7
Bovend’Eerdt et al., 2010 [50]	Y	Y	Y	Y	N	N	N	Y	Y	Y	Y	7
Braun et al., 2012 [52]	Y	Y	Y	Y	N	N	Y	Y	Y	Y	Y	8
Wang et al., 2023 [54]	Y	Y	Y	Y	Y	Y	Y	Y	N	Y	Y	9
Park et al., 2015 [51]	Y	Y	N	Y	N	N	N	Y	N	Y	Y	5
Wan Liu et al., 2024 [55]	Y	Y	N	Y	N	N	Y	N	N	Y	Y	5
Wang et al., 2020 [53]	Y	Y	N	Y	N	N	Y	Y	N	N	Y	5

Note: Y = criterion fulfilled; N = criterion not fulfilled.

## Data Availability

All data extracted from the included trials and used in this meta-analysis are openly available in the Supplementary Excel File provided with this manuscript. Statistical analyses were conducted in R software (R Foundation for Statistical Computing, Vienna, Austria; version 2024.12.0.467) using the metafor and orchaRd packages. The complete R code applied for the analyses is also included in the Supplementary Materials to ensure full reproducibility.

## Supplementary Materials

The following supporting information can be downloaded at: https://www.mdpi.com/article/10.3390/medicina62010174/s1, Supplementary Material S1: full search log and database queries, screening records, extraction templates, and statistical modeling R scripts.
