# Supplementary material for: Stroke Neurorehabilitation and the Role of Motor Imagery Training: Do ARAT and Barthel Index Improvements Support Its Clinical Use? A Systematic Review and Meta-Analysis
Source: Medicina (Kaunas). 2026 Jan 15;62(1):174. doi: 10.3390/medicina62010174 (PMC12843224; doi:10.3390/medicina62010174)
Supplement: Supplementary file 1 [file medicina-62-00174-s001.zip › Supplementary Material 1. Search database.pdf]

Supplementary Material 1: Database formulas during literature search (25/07/2025)

PUBMED Search Formula: 581

("motor imagery training"[All Fields] OR "mental practice"[All Fields] OR "mental imagery"[All Fields] OR "motor imagination"[All Fields] OR "mental rehearsal"[All Fields] OR "motor imagery"[All Fields] OR "Visual imagery"[All Fields] OR "imagined movement"[All Fields] OR "kinaesthetic imagery"[All Fields] OR "kinesthetic imagery"[All Fields] OR "Visual imagery"[All Fields] OR ("kinaesthetic"[All Fields] OR "kinaesthetically"[All Fields] OR "kinaesthetics"[All Fields] OR "kinesthetic"[All Fields] OR "kinesthetically"[All Fields] OR "kinesthetics"[All Fields]) OR ("imageries"[All Fields] OR "imagery, psychotherapy"[MeSH Terms] OR ("imagery"[All Fields] AND "psychotherapy"[All Fields]) OR "psychotherapy imagery"[All Fields] OR "imagery"[All Fields])) AND ("stroke"[All Fields] OR "cerebrovascular accident"[All Fields] OR "brain infarction"[All Fields] OR "cerebral infarct"[All Fields] OR "cerebral hemorrhage"[All Fields] OR "poststroke"[All Fields] OR "post-stroke"[All Fields] OR "apoplexy"[All Fields] OR "cerebrovascular stroke"[All Fields] OR "CVA"[All Fields] OR "Brain stroke"[All Fields] OR "vascular accident"[All Fields] OR "stroke rehabilitation"[All Fields] OR "stroke recovery"[All Fields] OR "ischemic"[All Fields] OR "hemorrhagic"[All Fields] OR ("hemiplegia"[MeSH Terms] OR "hemiplegia"[All Fields] OR "hemiplegias"[All Fields]) OR "Unilateral paralysis"[All Fields]) AND ("randomized controlled trial"[All Fields] OR "randomised controlled trial"[All Fields] OR "clinical trial"[All Fields] OR "controlled clinical trial"[All Fields] OR "RCT"[All Fields] OR "intervention study"[All Fields] OR "randomized"[All Fields] OR "randomised"[All Fields] OR ("random allocation"[MeSH Terms] OR ("random"[All Fields] AND "allocation"[All Fields]) OR "random allocation"[All Fields] OR "randomization"[All Fields] OR "randomized"[All Fields] OR "random"[All Fields] OR "randomisation"[All Fields] OR "randomisations"[All Fields] OR "randomise"[All Fields] OR "randomised"[All Fields] OR "randomising"[All Fields] OR "randomizations"[All Fields] OR "randomize"[All Fields] OR "randomizes"[All Fields] OR "randomizing"[All Fields] OR "randomness"[All Fields] OR "randoms"[All Fields]) OR ("clinical trials as topic"[MeSH Terms] OR ("clinical"[All Fields] AND "trials"[All Fields] AND "topic"[All Fields]) OR "clinical trials as topic"[All Fields] OR "trial"[All Fields] OR "trial s"[All Fields] OR "trialed"[All Fields] OR "trialing"[All Fields] OR "trials"[All Fields]) OR ("group s"[All Fields] OR "grouped"[All Fields] OR "grouping"[All Fields] OR "groupings"[All Fields] OR "groups s"[All Fields] OR "population groups"[MeSH Terms] OR ("population"[All Fields] AND "groups"[All Fields]) OR "population groups"[All Fields] OR "group"[All Fields] OR "social group"[MeSH Terms] OR ("social"[All Fields] AND "group"[All Fields]) OR "social group"[All Fields] OR "groups"[All Fields]))

Web of Science Search Formula: 1113

("motor imagery training" OR "mental practice" OR "mental imagery" OR "motor imagination" OR "mental rehearsal" OR "motor imagery" OR "Visual imagery" OR "imagined movement" OR "kinaesthetic imagery" OR "kinesthetic imagery" OR "visual imagery" OR kinesthetic OR kinaesthetic OR imagery) (Topic) AND ("stroke" OR "cerebrovascular accident" OR "brain infarction" OR "cerebral infarct" OR "cerebral hemorrhage" OR "poststroke" OR "post-stroke" OR "apoplexy" OR "cerebrovascular stroke" OR "CVA" OR " Brain stroke " OR "vascular accident" OR "stroke rehabilitation" OR "stroke recovery" OR "ischemic" OR "hemorrhagic" OR Hemiplegia OR “Unilateral paralysis”) (Topic) AND ("randomized controlled trial" OR "randomised controlled trial" OR "clinical trial" OR "controlled clinical trial" OR "RCT" OR "intervention study" OR

"randomized" OR "randomised" OR Random OR TRIAL OR GROUP) (Topic) and Web of Science Core Collection (Database)

COCHRANE Library Search Formula: 507

(‘motor imagery training’ OR ‘mental practice’ OR ‘mental imagery’ OR ‘motor imagination’ OR ‘mental rehearsal’ OR ‘motor imagery’ OR ‘Visual imagery’ OR ‘imagined movement’ OR ‘kinaesthetic imagery’ OR ‘kinesthetic imagery’ OR ‘visual imagery’ OR kinesthetic OR kinaestheti OR imagery) AND (“stroke” OR ‘cerebrovascular accident’ OR ‘brain infarction’ OR ‘cerebral infarct’ OR ‘cerebral haemorrhage’ OR ‘poststroke’ OR ‘post-stroke’ OR ‘apoplexy’ OR ‘cerebrovascular stroke’ OR ‘CVA’ OR ‘Brain stroke’ OR ‘vascular accident’ OR ‘stroke rehabilitation’ OR ‘stroke recovery’ OR “ischemic” OR ‘haemorrhagic’ OR Hemiplegia OR ‘Unilateral paralysis’) AND (‘randomised controlled trial’ OR ‘randomised controlled trial’ OR ‘clinical trial’ OR ‘controlled clinical trial’ OR ‘RCT’ OR ‘intervention study’ OR “randomised” OR ‘randomised’ OR Random OR trial OR group) in Title Abstract Keyword.

ScienceDirect Search Formula: 756

TITLE-ABS-KEY("motor imagery " OR "mental imagery" OR Visual imagery" OR " kinaesthetic imagery" OR "visual imagery") AND ("stroke" OR "cerebrovascular accident" OR "brain infarction" OR "cerebral infarct)

Scopus Search Formula: 846

TITLE-ABS-KEY(("motor imagery training" OR "mental practice" OR "mental imagery" OR "motor imagination" OR "mental rehearsal" OR "motor imagery" OR "Visual imagery" OR "imagined movement" OR "kinaesthetic imagery" OR "kinesthetic imagery" OR "visual imagery" OR kinesthetic OR kinaestheti OR imagery) AND ("stroke" OR "cerebrovascular accident" OR "brain infarction" OR "cerebral infarct" OR "cerebral hemorrhage" OR "poststroke" OR "post-stroke" OR "apoplexy" OR "cerebrovascular stroke" OR "CVA" OR " Brain stroke " OR "vascular accident" OR "stroke rehabilitation" OR "stroke recovery" OR "ischemic" OR "hemorrhagic" OR Hemiplegia OR "Unilateral paralysis") AND ("randomized controlled trial" OR "randomised controlled trial" OR "clinical trial" OR "controlled clinical trial" OR "RCT" OR "intervention study" OR "randomized" OR "randomised" OR Random OR TRIAL OR GROUP))
